# A case of primary lung adenocarcinoma mimicking metastatic papillary thyroid carcinoma

**DOI:** 10.1111/1759-7714.15194

**Published:** 2023-12-22

**Authors:** Akira Tanaka, Riki Okita, Takushi Morishige, Masanori Okada, Hidetoshi Inokawa, Katsutoshi Hirazawa, Kaori Kameyama, Akihiko Ikeda, Eiji Ikeda

**Affiliations:** ^1^ Department of Pathology Yamaguchi University Graduate School of Medicine Yamaguchi Japan; ^2^ Department of Thoracic Surgery National Hospital Organization Yamaguchi Ube Medical Center Yamaguchi Japan; ^3^ Breast and Gastrointestinal Surgery National Hospital Organization Yamaguchi Ube Medical Center Yamaguchi Japan; ^4^ Department of Diagnostic Pathology Showa University Northern Yokohama Hospital Yokohama Japan; ^5^ Department of Surgery Shunan Memorial Hospital Yamaguchi Japan; ^6^ Department of Clinical Research National Hospital Organization Yamaguchi Ube Medical Center Yamaguchi Japan

**Keywords:** lung adenocarcinoma, papillary thyroid carcinoma, RET‐GOLGA5 fusion gene

## Abstract

A 61‐year‐old woman, who had a history of total thyroidectomy for follicular variant of papillary thyroid carcinoma (PTC), visited our hospital for assessment of an enlarging nodule which appeared in the lung with multiple metastatic lesions of PTC which had been stable for 17 years. Wedge resection of the lung was performed. Miliary nodules were confirmed to be metastatic PTCs based on their morphological as well as immunohistochemical findings. As for the main nodule, its morphological features suggested a diagnosis of metastatic PTC, while its immunohistochemical findings were identical with primary lung adenocarcinoma. Further genetic analysis provided no definitive information for the diagnosis of the main nodule. The present case shows the need of comprehensive analyses for differentiation between primary lung adenocarcinoma and metastatic PTCs.

## Graphical abstract

We report a case of primary lung adenocarcinoma arising in the lung with multiple metastatic nodules of the follicular variant of papillary thyroid carcinoma (PTC). This case highlights the need for comprehensive analyses including morphology, immunohistochemistry and genetic analyses to differentiate the primary lung adenocarcinoma from the metastatic PTCs.

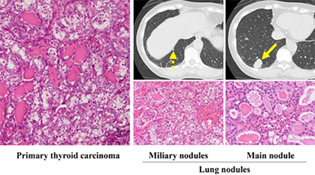

## INTRODUCTION

Patients with papillary thyroid carcinoma (PTC) often develop multiple lung metastases. There are cases in which lung nodules of patients with PTC need to be resected for differential diagnosis between the metastatic PTC and primary lung cancer, in order to determine the therapeutic protocol. In general, the metastatic PTC grows quite slowly when compared with primary lung cancer with more aggressive behavior. Here, we present a case of primary lung adenocarcinoma which appeared in the lung with multiple nodules of metastatic PTC and showed the morphological features of PTC.

## CASE REPORT

**FIGURE 1:**
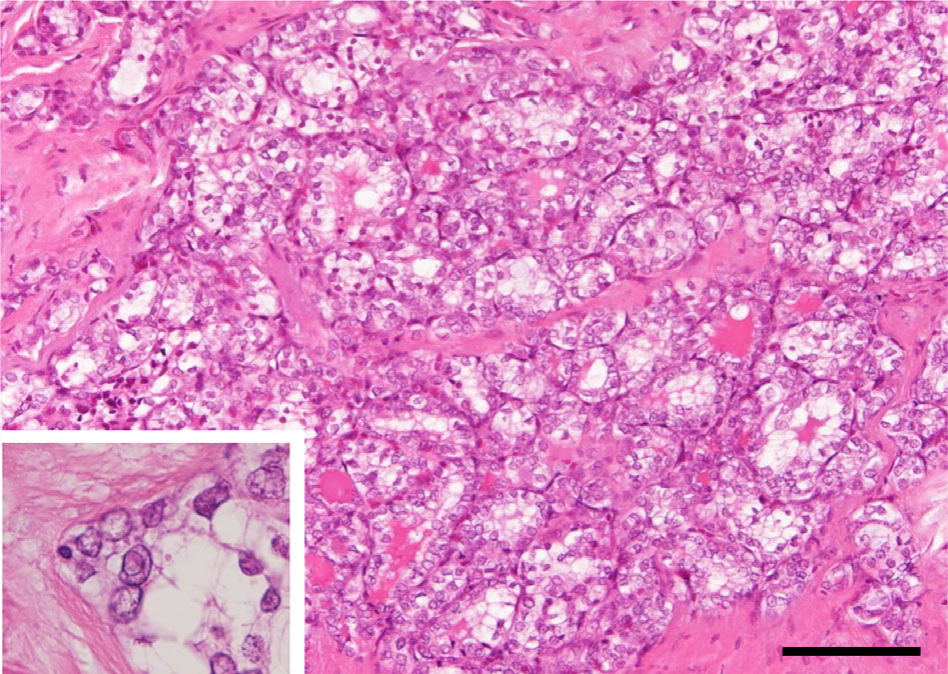
Morphological findings of primary thyroid carcinoma. Tumor cells grow forming a follicular pattern containing colloid material. A papillary growth pattern was undetectable. The presence of cells with intranuclear cytoplasmic inclusion and nuclear groove are confirmed (inset). Scale bar, 100 μm.

**FIGURE 2:**
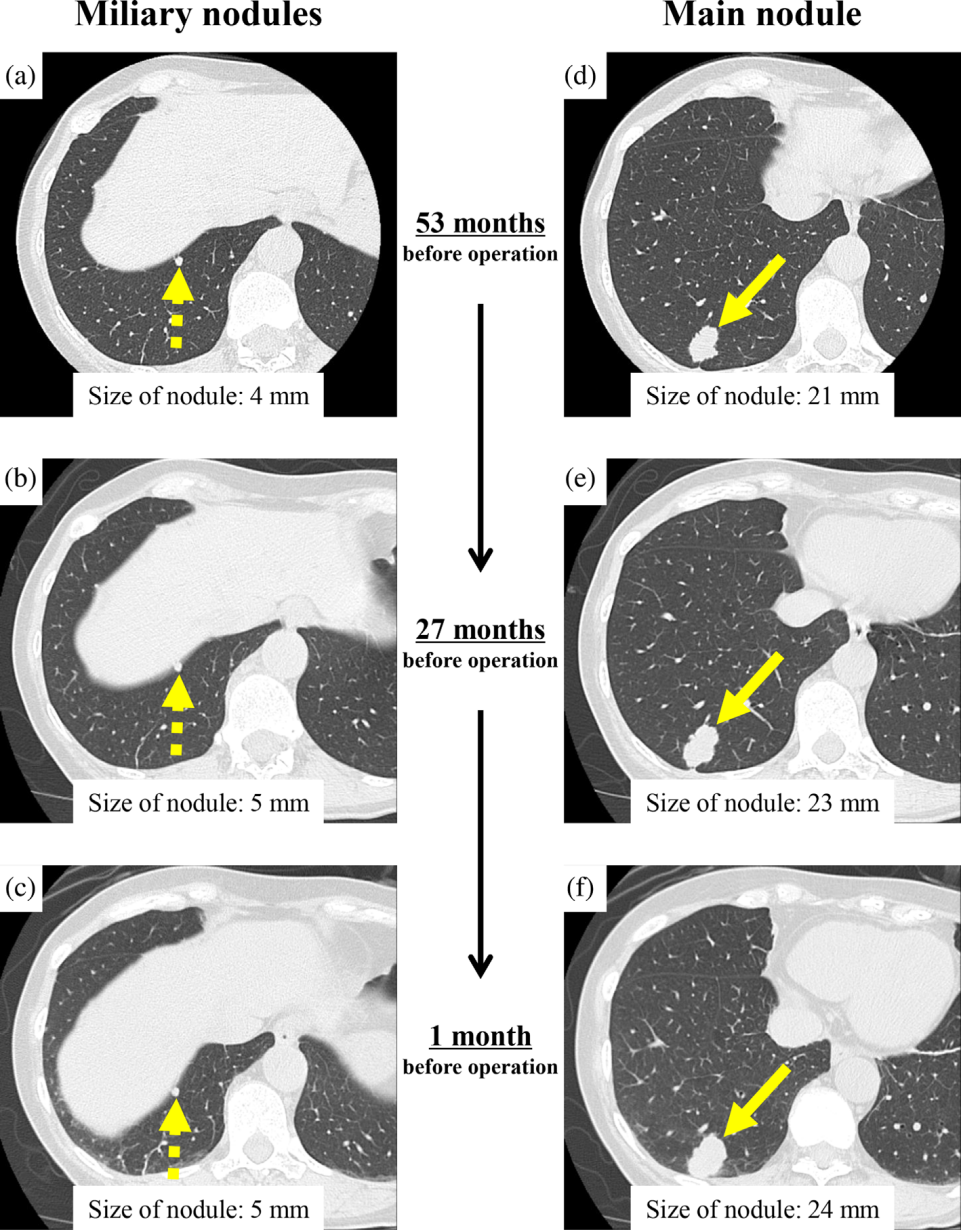
Computed tomography (CT) scan findings of lung nodules. (a–c) Multiple miliary nodules followed up for 17 years were found (dashed arrow). (d–f) A main nodule showing slow enlargement was detected in the right lower lobe (solid arrow). Chronological CT scans at 53 months (a, d), 27 months (b, e) and 1 month (c, f) before the operation demonstrate miliary nodules (a–c) remained stable, while the main nodule (d–f) was slowly enlarging.

A 61‐year‐old woman, who had a history of total thyroidectomy, visited our hospital for assessment of an enlarging nodule in the lung with multiple stable miliary nodules which had been followed up under the clinical diagnosis of metastatic PTCs. Seventeen years before this visit, she underwent total thyroidectomy when histological examination revealed a thyroid tumor which comprised cells growing in a follicular pattern, and cells with intranuclear cytoplasmic inclusion and nuclear groove were detected (Figure 1). The tumor was diagnosed as a follicular variant of PTC. Postoperatively, she received two iodine‐131 internal irradiations. Multiple lung miliary nodules have since been followed up under the diagnosis of metastatic PTC and have remained stable on successive CT scans for 17 years (Figure 2a–c). During the follow‐up, a nodule showing slow enlargement appeared in the right lower lobe (Figure 2d–f), and lung wedge resection was performed.

**FIGURE 3:**
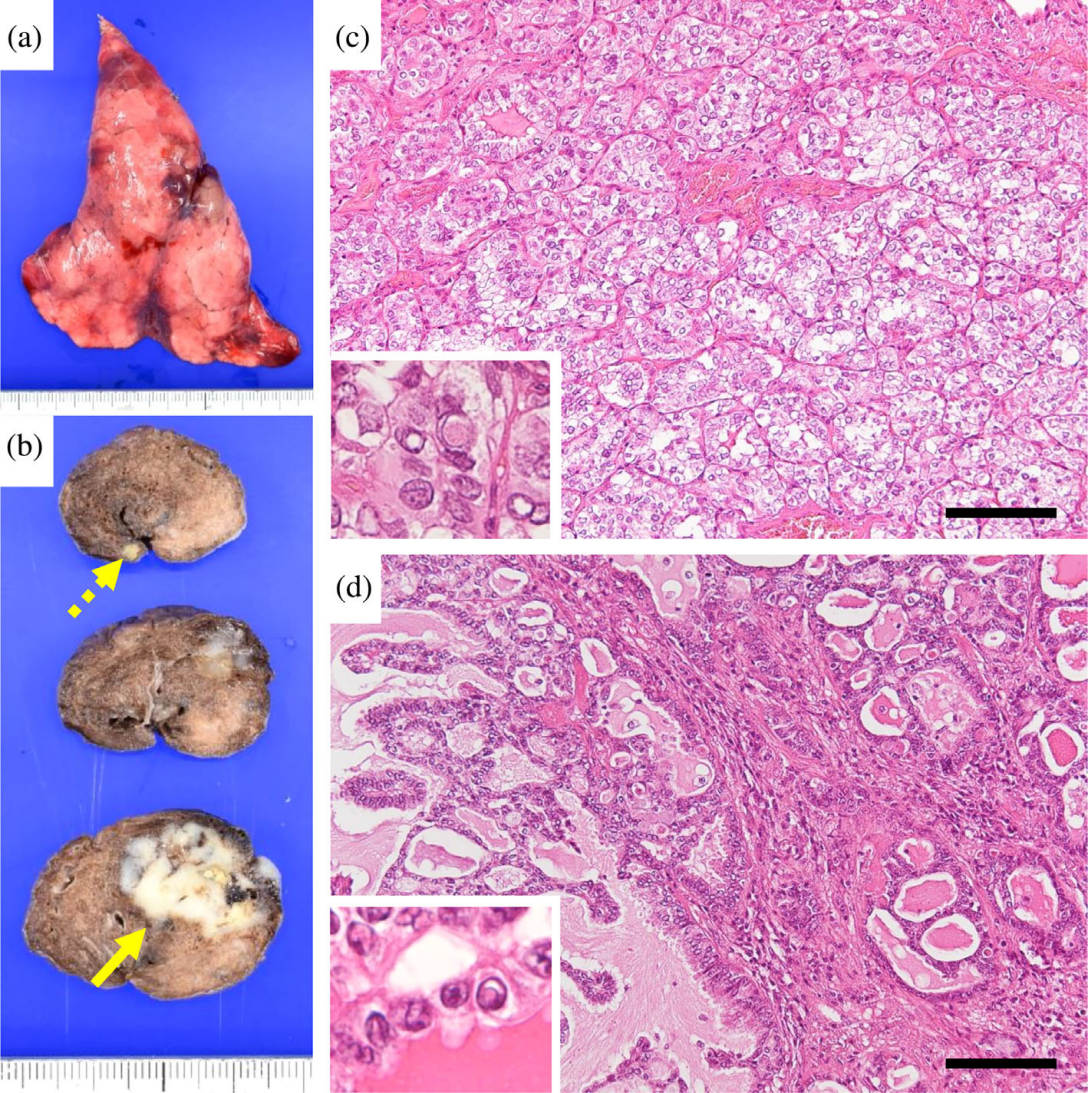
Morphological findings of lung miliary nodules and the main enlarging nodule. (a, b) Cut surfaces (b) of the wedge‐resected lung (a) show the presence of miliary nodules (dashed arrow) as well as the main nodule (solid arrow). (c) The miliary nodules were comprised of tumor cells growing in a follicular pattern containing colloid material, and the presence of cells with intranuclear cytoplasmic inclusion and nuclear groove are confirmed (inset). Thus, miliary nodules are identical morphologically with the primary thyroid tumor. (d) In the main nodule, tumor cells can be seen growing in tubular and papillary patterns with some follicle‐like tubules containing colloid‐like material. The presence of cells with intranuclear cytoplasmic inclusion and nuclear groove are confirmed (inset). Morphological features of the main nodule suggest a diagnosis of metastatic papillary thyroid carcinoma (PTC) with a papillary pattern. Scale bars in (c) and (d), 100 μm.

**FIGURE 4:**
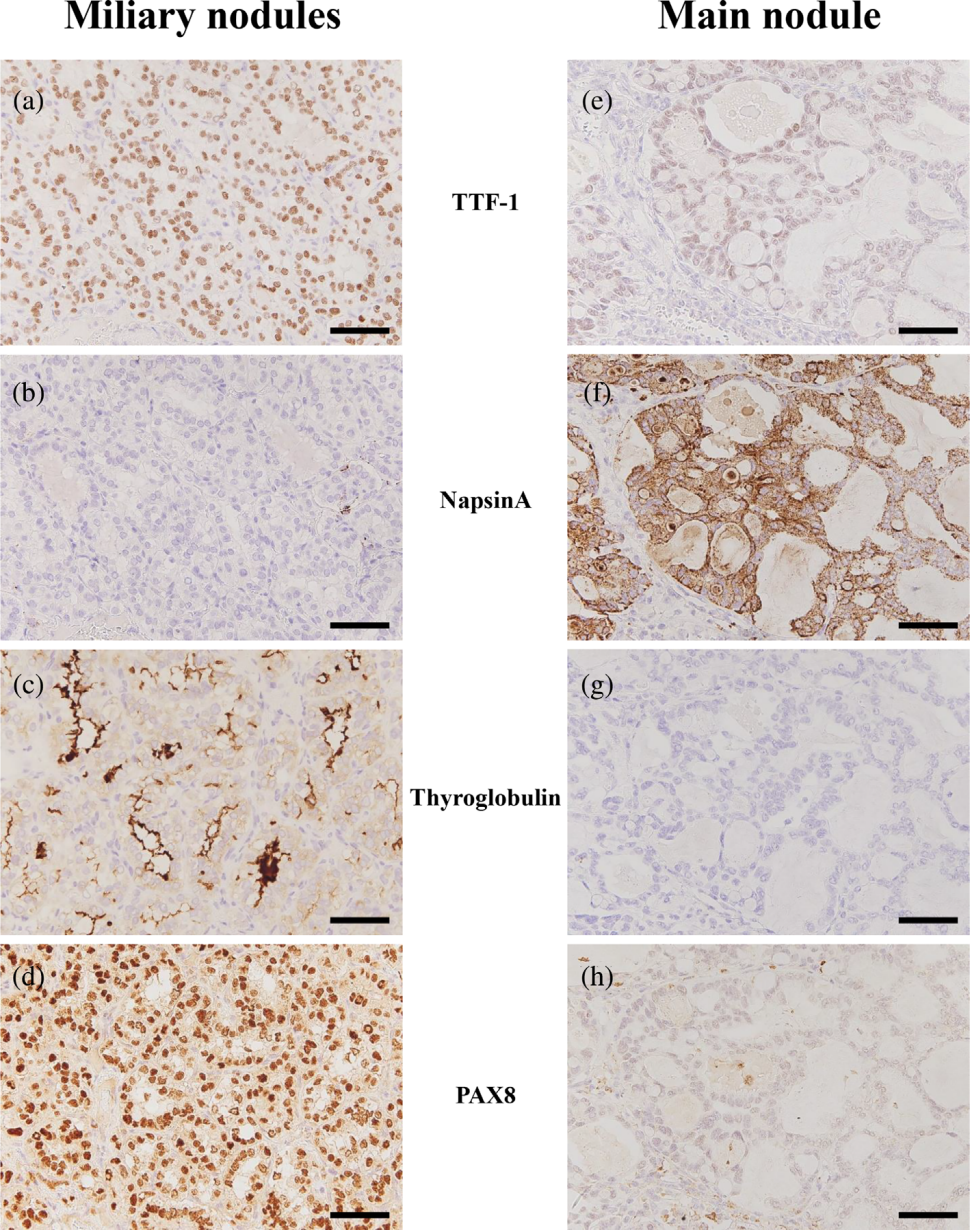
Immunohistochemical findings of miliary nodules and the main nodule. (a–d) Tumor cells in miliary nodules were positive for thyroid transcription factor‐1 (TTF‐1) (a), thyroglobulin (c) and paired box 8 (PAX8) (d), but negative for napsin‐A (b). (e–h) Tumor cells in the main nodule were positive for TTF‐1 (e) and napsin A (f), but negative for thyroglobulin (g) and PAX8 (h). Thus, immunohistochemically, the miliary nodules and the main nodule were identical with metastatic PTCs and primary lung adenocarcinoma, respectively. Scale bars in (a–h), 50 μm.

The main nodule (25 × 25 mm) and smaller miliary nodules exist in the resected lung (Figure 3a,b). The miliary nodules showed the identical morphology to that of the primary PTC of follicular variant (Figure 3c). The main nodule comprised tumor cells forming tubular and papillary structures with some follicle‐like tubules containing colloid‐like material (Figure 3d). A lepidic growth pattern was undetectable. Cells with intranuclear cytoplasmic inclusion and nuclear grooves were observed (Figure 3d, inset). Despite some unusual characteristics for PTC, morphological features of the main nodule suggested the diagnosis of a metastatic PTC with papillary growth pattern. Immunohistochemically, cells in miliary nodules were positive for thyroid transcription factor‐1 (TTF‐1), thyroglobulin and paired box 8 (PAX8), but negative for napsin‐A, while cells in the main nodule were positive for TTF‐1 and napsin A, but negative for thyroglobulin and PAX8 (Figure 4). Thus, the miliary nodules were identical with metastatic PTCs both morphologically and immunohistochemically.^1^ In contrast, the main nodule with morphological features of PTC showed the immunophenotype identical with primary lung adenocarcinoma.^1^ Oncomine Dx target test revealed the RET‐GOLGA5 fusion gene in cells of the main nodule, but no RET fusion genes were detected in the primary thyroid carcinoma. The patient was alive with multiple lung nodules 19 months after lung resection. Written informed consent was obtained from the patient to use her clinical information for research purposes.

## DISCUSSION

In the case reported here, a main enlarging nodule was found in the resected lung with multiple miliary nodules. Miliary nodules can be easily diagnosed as metastatic PTCs since both morphological and immunohistochemical findings were identical with the follicular variant of PTC. As for the main nodule, its morphological findings rather suggest a diagnosis of metastatic PTC although with a papillary growth pattern which had not been detected in the primary thyroid carcinoma, while its immunohistochemical findings were identical with primary lung adenocarcinoma. Oncomine Dx target test demonstrated that RET‐GOLGA5 fusion gene was found in the enlarging nodules but not in the primary thyroid carcinoma. Considering that RET‐GOLGA5 fusion gene is reported to be quite rare in both thyroid cancer and non‐small cell lung cancer,^2^, ^3^, ^4^, ^5^ it is hard to say whether the presence of RET‐GOLGA5 fusion gene in the main nodule of the present case supports the diagnosis of primary lung adenocarcinoma. RET‐GOLGA5 fusion gene was first reported in pediatric patients with PTC who had been exposed in their childhood to radioiodine released from the Chernobyl reactor.^6^, ^7^ Together with a reported in vitro study demonstrating that RET‐GOLGA5 fusion gene accelerates the cell proliferation by activating AKT, ERK, S6, and PLCy‐1,^8^ it remains possible that RET‐GOLGA5 fusion gene in the main enlarging nodule of the present case was acquired by internal irradiation therapy with radioiodine after thyroidectomy, enhancing the growth rate as well as modifying epigenetically the morphology and immunophenotype of one of the metastatic nodules of PTC. Unfortunately, the Oncomine DX target test failed to determine the status of the RET‐fusion genes in miliary nodules due to the small sample volume.

Recently, Takemura et al. reported a case with rapidly growing lung lesions which were diagnosed as metastatic PTC based on the detection of RET‐NCOA4 fusion gene in both the PTC primary lesion and a nodule of pleural dissemination, although lung lesions showed not only the morphology but also the immunophenotypes consistent with primary lung adenocarcinoma.^9^ In the present case, giving priority to the immunophenotype over the results of the other analyses, we diagnosed the main nodule as primary lung adenocarcinoma, although its growth rate was relatively slow for primary adenocarcinoma without a lepidic growth pattern. Thus, comprehensive analyses^10^ are required for differentiation between primary lung adenocarcinoma and metastatic PTCs. Accumulation of histological studies on metastatic PTC in lungs is required in order to establish the appropriate diagnostic protocol for the management of patients with PTC.

## AUTHOR CONTRIBUTIONS

Akira Tanaka: Investigation (pathological diagnosis), resources, visualization, writing‐original draft preparation. Riki Okita: Conceptualization, data curation, investigation, resources, writing‐original draft preparation. Takushi Morishige: Investigation (pathological diagnosis), resources, writing‐review and editing. Masanori Okada: Data curation, resources, writing‐review and editing. Hidetoshi Inokawa: Data curation, resources, writing‐review and editing. Katsutoshi Hirazawa: Data curation, resources, writing‐review and editing. Kaori Kameyama: investigation (pathological diagnosis), resources, writing‐review and editing. Akihiko Ikeda: Data curation, resources, writing‐review and editing. Eiji Ikeda: Conceptualization, investigation (pathological diagnosis), resources, supervision, writing‐original draft preparation.

## CONFLICT OF INTEREST STATEMENT

The authors declare that they have no conflict of interest.
